# Glutamine Protects against Mouse Abdominal Aortic Aneurysm through Modulating VSMC Apoptosis and M1 Macrophage Activation

**DOI:** 10.7150/ijms.96395

**Published:** 2024-05-19

**Authors:** Jinxi Wang, Xingwen Da, Yifei Chen, Ancai Yuan, Jun Pu

**Affiliations:** Department of Cardiology, Renji Hospital, School of Medicine, State Key Laboratory for Oncogenes and Related Genes, Shanghai Cancer Institute, Shanghai Jiao Tong University, 160 Pujian Road, Shanghai 200127, China.

**Keywords:** Glutamine, Abdominal aortic aneurysm, Apoptosis, ROS, MMP

## Abstract

Glutamine (Gln), known as the most abundant free amino acid, is widely spread in human body. In this study, we demonstrated the protective effects of glutamine against mouse abdominal aortic aneurysm (AAA) induced by both angiotensin II (AngII) and calcium phosphate (Ca_3_(PO_4_)_2_) *in vivo*, which was characterized with lower incidence of mouse AAA. Moreover, histomorphological staining visually presented more intact elastic fiber and less collagen deposition in abdominal aortas of mice treated by glutamine. Further, we found glutamine inhibited the excessive production of reactive oxide species (ROS), activity of matrix metalloproteinase (MMP), M1 macrophage activation, and apoptosis of vascular smooth muscle cells (VSMCs) in suprarenal abdominal aortas of mice, what's more, the high expressions of MMP-2 protein, MMP-9 protein, pro-apoptotic proteins, and IL-6 as well as TNF-α in protein and mRNA levels in cells treated by AngII were down-regulated by glutamine. Collectively, these results revealed that glutamine protected against mouse AAA through inhibiting apoptosis of VSMCs, M1 macrophage activation, oxidative stress, and extracellular matrix degradation.

## Introduction

AAA, a lethal cardiovascular disease most commonly defined as a maximum infrarenal abdominal aortic diameter of 30mm or more, is asymptomatic early, leading to delayed diagnosis and final sudden death by rupture 1,2. Currently, clinic treatment for AAA is restricted to open surgery and interventional surgery and lack of pharmacological treatment approved by US Food and Drug administration 3,4. Therefore, identification of potentially effective drugs that can alleviate the formation and progression of AAA has been a research focus of our laboratory for a long time.

Glutamine was considered beneficial in anti-inflammation, anti-oxidation, and anti-apoptosis 5-7. Emerging studies found the close relationship between glutamine and cardivascular physiology and pathology 8. High glutamine concentrations promoted survival rate of cardiac progenitor cells and proliferation rate of vascular smooth muscle cells 9,10, and high intake of glutamine by humans was associated with lower risk of mortality, especially caused by cardiovascular diseases 11. Glutamine is a major source of energy for metabolism in cells and tissues. The metabolic pathway of glutamine begins with deamination by glutaminase to produce glutamate and ammonia, and further glutamate is converted into α-ketoglutaric acid (α-KG), an intermediate product of the tricarboxylic acid (TCA) cycle, which produces ATP. Our laboratory previously reported supplementation of α-KG as an effective therapeutic treatment for AAA 12. While as the precursor of α-KG, the direct effect of supplementation of glutamine on AAA has not been reported.

VSMCs, the most abundant cell type in the arterial wall, have many important functions including contractility, proliferation, phenotypic switches, and extracellular matrix regeneration 14. MMP has long been thought to play a central regulatory role in development, progression, and eventual rupture of AAA, since it promoted the degradation of extracellular matrix (ECM), especially among which the rupture of elastic fiber led to the structure destruction of abdominal aorta, finally causing sudden rupture of the aneurysm and life-threatening massive bleeding 15,16. Additionally, MMP itself could result in apoptosis of resident aortic cells and mass infiltration of inflammatory cells in aortic tissues 17. ROS in physiological levels played an important role in maintaining normal vascular functions like VSMC contraction, while uncontrolled overproduction of ROS resulted in oxidative stress, causing vascular damage, the recruitment of inflammatory cells, and activation of MMP, finally leading to numerous vascular diseases including AAA 18,19. Additionally, the tissue of abdominal aorta aneurysm is characterized by the mass infiltration of inflammatory cells including macrophages, whose polarization secretes mass inflammatory factors involved in the whole development and final formation of AAA 20,21. Previous studies have demonstrated that, due to macrophage polarization, M1 macrophage-derived IL-6 and TNF-α were closely associated with the pathogenesis of AAA in both human and mice 22-24. Taken together, excessive apoptosis of VSMCs, secretion of IL-6 and TNF-α by M1 macrophage activation, oxidative stress by excess aggregation of ROS, and activation of MMP gradually lead to destruction of vessel structure, finally causing AAA 14,25-27. Identification of drugs effective in inhibiting apoptosis of VSMCs, macrophage polarization, MMP activity, oxidative stress, and aggregation of ROS in abdominal aorta is the key to treat AAA.

Our current study conducted a series of experiments, aiming to investigate whether supplementation of glutamine is effective in treating mouse AAA.

## Materials and Methods

### Animal studies

All animal experiments complied with the National Institutes of Health Guidelines on the Care and Use of Laboratory Animals (National Institutes of Health Publication, 8th edition, 2011), and approval was obtained from the Animal Ethics Committee of Renji Hospital, School of Medicine, Shanghai Jiao Tong University. AngII-induced or Ca_3_(PO_4_)_2_-induced AAA models were established as previously reported 12. The mice were housed in an environment with a 12h-light/12h-dark cycle, a temperature of 24 ± 2°C and the humidity maintained at 40 ± 5%, with free access to diet and water. All APOE-/- mice and C57BL/6J mice were purchased from Gempharmatech (China). To examine the effect of glutamine on AAA mouse models, L-Glutamine (1 g/kg body weight per day, A600224-0100; Sangon Biotech (Shanghai), China) or dissolved vehicle (Vehicle) was administrated intragastrically 13.

### Establishment of AngII-induced mouse AAA model

Male 6 to 8-week-old *Apoe^-/-^* mice were subcutaneously implanted with a mini-osmotic pump (Alzet model 2004, 28-day delivery; Durect Corporation, USA) loaded with AngII (1000 ng/kg/min, A9525; Sigma, MO, USA) or saline (0.9% NaCl) for 28 days with a high-fat diet. At 28 days after mini-pump implantation, mice were sacrificed to validate the AAA formation, and the maximum diameters of the abdominal aorta were measured using a measuring tape. The abdominal aorta was considered as aneurysm if the diameter increased ≥ 50% compared with the saline group. If any mouse died prior to the study endpoint, it would be subjected to autopsy.

### Establishment of Ca_3_(PO_4_)_2_-induced mouse AAA model

Under general anesthesia, infrarenal abdominal aortas of male 6 to 8-week-old C57BL/6J mice were isolated and incubated with 0.5 mol/L calcium chloride (10 min), followed by phosphate-buffered saline (PBS) (5 min) to form Ca_3_(PO_4_)_2_. Before suturing, the abdominal cavities were washed with saline (0.9% NaCl). At 28 days after surgery, the infrarenal abdominal aortas were collected for the quantification of the external diameters by a measuring tape.

### *In Vivo* Micro-Ultrasound Imaging

To exhibit the morphological features of abdominal aortas* in vivo*, the Micro-Ultrasound Imaging (MUI) was performed by a Vevo770 High-Resolution *In Vivo* Micro-ultrasound Imaging System (VisualSonics Inc., Ontario, Canada) with a 55-MHz transducer for mice. In accordance with the manufacturer's instructions, MUI images were acquired using the B mode and Color Mode. Three measurements of the external diameters of abdominal aortas on both short axis and long axis were performed by a blinded investigator.

### Histological analysis

Abdominal aortas isolated from mice were perfused with saline and fixed with 4% paraformaldehyde. Then, the aortas were embedded in paraffin, followed by serial sectioning. Paraffin-embedded sections (3-4 µm thick) were deparaffinized and prepared for hematoxylin and eosin (HE) staining to observe general morphology, Elastin Van Gieson (EVG) staining was performed to assess elastin degradation, and Masson's Trichrome staining was used to determine collagen content. The number of breaks per vessel was counted for the quantification of the elastin degradation. Fold changes of collagen content were calculated using Masson's Trichrome staining by ImageJ.

#### Immunofluorescence

For immunofluorescence staining, frozen aortic tissue sections and Raw264,7 growing on glass coverslipes with a proper density were fixed for 30 minutes, permeated for 30 minutes, and blocked for 1 hour, respectively. Then, the sections and glass coverslipes were incubated with anti-CD68 (1:100, HY-P80605, MedChemExpress, China) primary antibody overnight at 4°C. After washing with PBS for three times, Alex Fluor 488 conjugated secondary antibody (1:200, invitrogen, USA) were applied for 1 hour at 37°C in the dark. Sections and glass coverslipes were mounted with ProLong Gold anti-fade reagent with 4',6-diamidino-2-phenylindol (DAPI, P36931; Invitrogen, USA) for confocal microscopy (Leica TCS SP8; Leica, Germany).

### *In Situ* Dihydroethidium (DHE) staining

To determine the superoxide generation, we used *in situ* dihydroethidium (DHE) staining (S0063; Beyotime, China). Briefly, after perfused with saline, fresh abdominal aortas were snap-frozen into optimal cutting temperature (OCT) compound (4583; SAKURA Tissue-TEK, USA). Cryosections (5 µm thick) were incubated with DHE staining (red). A fluorescence microscope was used to capture images.

### Assay of Matrix Metalloproteinase activity

Cryosections of fresh abdominal aortas (10 µm thick) were used for MMP activity assays by* in situ* zymography. In brief, cryosections were incubated with a fluorogenic gelatin substrate (DQ gelatin) (E-12055; Molecular Probes, USA) in accordance with the manufacturer's protocols. MMP activity was shown as green fluorescence. Smooth muscle cells were co-stained with an anti-α-SMA antibody and nuclei were counterstained with DAPI.

MMP activity was also assessed by gelatin zymography, as previously described 12. Proteins in mouse aorta tissue homogenates, MOVAS, or RAW264.7 were electrophoresed in SDS-PAGE gels containing 0.8 mg/ml gelatin. After being washed in 2.5% Triton X-100, the gels were incubated in zymography buffer [50 mmol/L Tris (pH 8.0), 10 mmol/L CaCl_2_, and 0.05% Brij 35] for 48 h (37°C), followed by Coomassie brilliant blue staining.

### Assessment of cell apoptosis

Terminal deoxynucleotidyl transferase dUTP nick-end labeling (TUNEL) assay was performed to estimate cell apoptosis in abdominal aortas by an* in situ* Cell Death Detection Kit (11684817910; Roche, Germany). Smooth muscle cells were stained by an anti-αSMA antibody and nuclei were stained by DAPI. After visualized by a confocal microscope, the ratio of TUNEL-positive nuclei to total nuclei was calculated.

### Cell culture and treatment

Mouse macrophage cell line (RAW264.7 cells, ATCC, Manassas, VA, USA) and mouse vascular smooth muscle cell line (MOVAS cells, ATCC) were cultured in high glucose Dulbecco's Modified Eagle Medium (DMEM, Hyclone, Logan, UT, USA) supplemented with 10 % fetal bovine serum and 1% penicillin/streptomycin in a humidified 95 % air and 5 % CO2 atmosphere at 37°C, with medium being changed every 2-3 days. The cells were plated on 60-mm culture dishes at a density of 2×10^5^ cells/ml, permitted to adhere in complete medium for 24 h, and changed to serum-free medium (RAW264.7 cells) or 1 % serum medium (MOVAS cells) to serum starve for about 8 hours, then cells from passages were pretreated with vehicle or glutamine (10 mmol/L), followed by stimulation with saline or AngII (100 nmol/L) for 48 h and finally harvested for further analysis.

### Protein extraction and western blot

Proteins were extracted from MOVAS and RAW264.7. The separation of proteins was performed on 10%-12.5% SDS-PAGE gels and the proteins were transferred to a nitrocellulose membrane. The membranes were blocked with protein-free rapid blocking buffer (PS108P; Epizyme, China) and incubated at 4°C overnight with primary antibodies against MMP-2 (1:1000, 66366-1-Ig; Proteintech, USA), MMP-9 (1:1000, A5725; Biomake, China); β-actin (1:4000, 81115-1-RR; Proteintech, USA), Caspase-3 (1:1000, 19677-1-AP; Proteintech, USA), Cleaved Caspase-3 (1:1000, 25128-1-AP; Proteintech, USA), Bcl2 (1:1000, AF6285; Beyotime, China), Bax (1:1000, AF5120; Beyotime, China), IL-6 (1:1000, 26404-1-AP; Proteintech, USA), TNF-α (1:1000, 26162-1-AP; Proteintech, USA). After the incubation of corresponding secondary antibodies (1:10000, Jackson ImmunoResearch Laboratories, USA), enhanced chemiluminescence (Share-bio, China) was used to show the protein bands with the ImageQuant LAS 4000 mini detection system (General Electric Co.).

### RNA isolation and Real-Time quantitative PCR (RT-qPCR)

TRIzol was applied to isolate total RNA which is used for cDNA synthesis with a PrimeScript RT reagent kit (Takara, Japan). The mRNA expression was detected by Real-time quantitative PCR with SYBR Premix Ex Taq™ (Takara, Japan) using a LightCycler® 480 Realtime PCR System (Roche Applied Science). β-actin was used as the housekeeping gene. Primer information is shown in Table 1.

### Statistical Analysis

Continuous variables are shown as mean±SD, and categorical variables are presented as number (percentage). Normality of data distribution was assessed using the Shapiro-Wilk test and the equality of variances was determined using the Levene test. For 2 groups comparisons, the Student's t test (equal variances) or unequal variance t test (unequal variances) was applied for normally distributed variables, otherwise the Mann-Whitney U test was performed. In comparisons involving more than two groups, a two-way ANOVA followed by Bonferroni post hoc analysis was employed. The Chi-squared test or the Fisher's exact test was used for the bivariate comparisons of categorical variables. Statistical analysis was performed with SPSS software version 26.0 and GraphPad Prism 8. A value of P<0.05 was considered statistically significant.

## Results

### Glutamine limited the formation of mouse AAA induced by AngII

**Figure 1:**
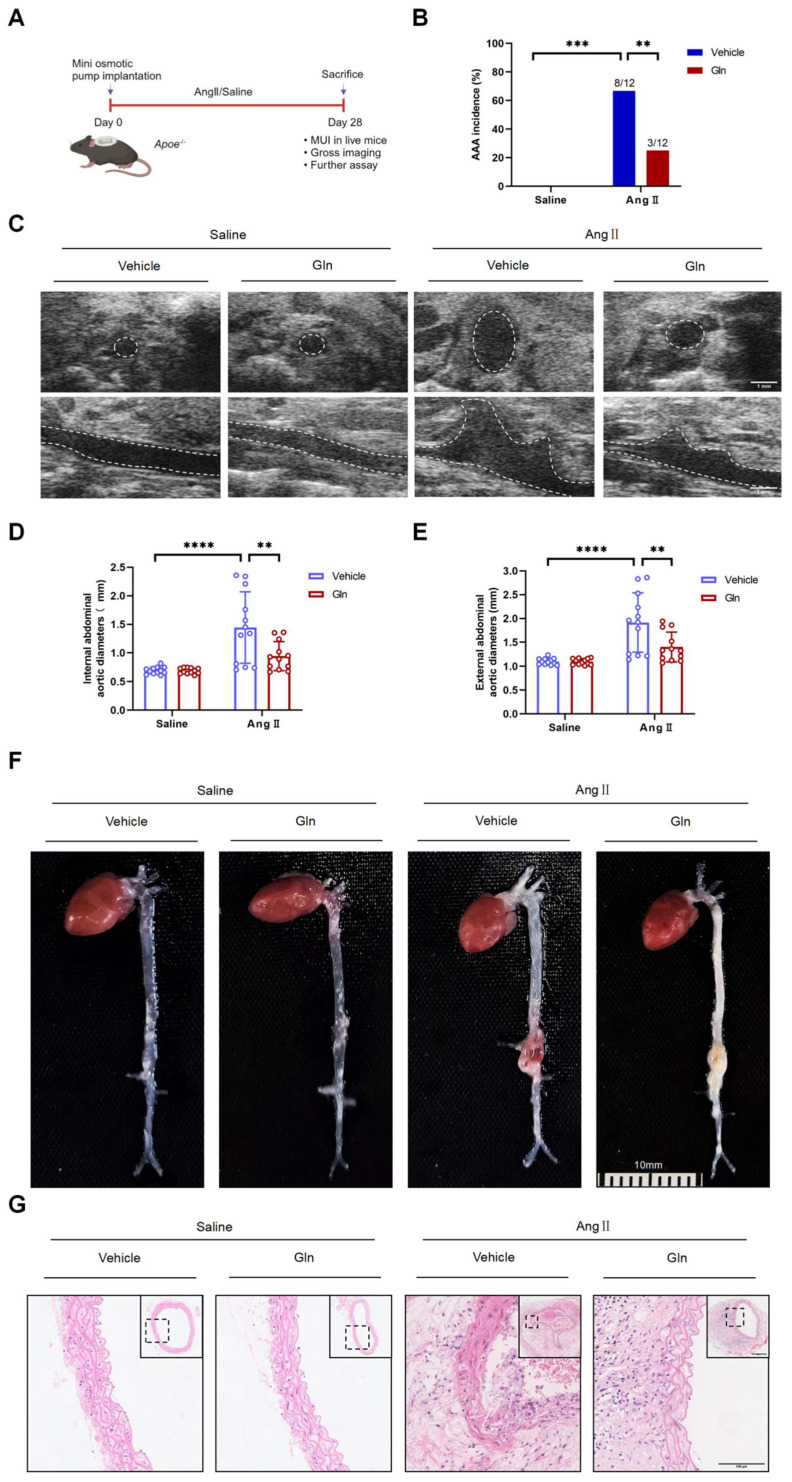
**Glutamine limited the formation of mouse AAA induced by AngII.** A, Schematic protocol: ApoE^-/-^ mice were subcutaneously injected with saline or AngII by a mini osmotic pump for 28 days (n=12 per group), then ApoE^-/-^ mice were sacrificed. (The image was created with BioRender.com) B, The incidence of AngII-induced AAA in indicated groups (n=12 per group). Data were analyzed by a Fisher exact test. **P<0.01; ***P<0.001. C, Representative images of abdominal aortas visualized by MUI using the B mode in indicated groups. D and E, Quantification of the internal diameter of suprarenal abdominal aortas measured by MUI and external diameter by a Digital Vernier Caliper in indicated groups (n=12 per group). Data were analyzed by 2-way ANOVA followed by the Bonferroni post hoc test. ***P<0.001; ****P<0.0001. F, Representative images of the macroscopic features of AAA formation in indicated groups. G, Representative images of HE staining in suprarenal abdominal aortas in indicated groups. Data are expressed as mean ± SD. Abbreviations: AngII, angiotensin II; Gln, glutamine; AAA, abdominal aortic aneurysm; MUI, micro-ultrasound imaging; HE staining, hematoxylin and eosin staining.

To examine whether glutamine limited the formation of mouse AAA, we constructed AngII-induced mouse AAA model (Figure 1A). Compared with Vehicle treatment, intragastric administration of l-glutamine significantly attenuated AAA incidence in AngII-induced AAA model (Figure 1B). Moreover, decreased internal and external diameter (Figure 1D and 1E) of suprarenal abdominal aortas were observed after glutamine treatment compared with vehicle treatment in AngII-induced AAA model, as shown by *in vivo* micro-ultrasound imaging (MUI) (Figure 1C) and measuring tape (Figure 1F). More complete aortic tissue, smaller cell nucleus and weaker staining in cell nucleus were seen by glutamine treatment compared with vehicle treatment in AngII-induced AAA model (Figure 1G).

### Glutamine's protective effects above on AngII-induced mouse AAA model were also seen in mouse AAA model by Ca_3_(PO_4_)_2_

**Figure 2:**
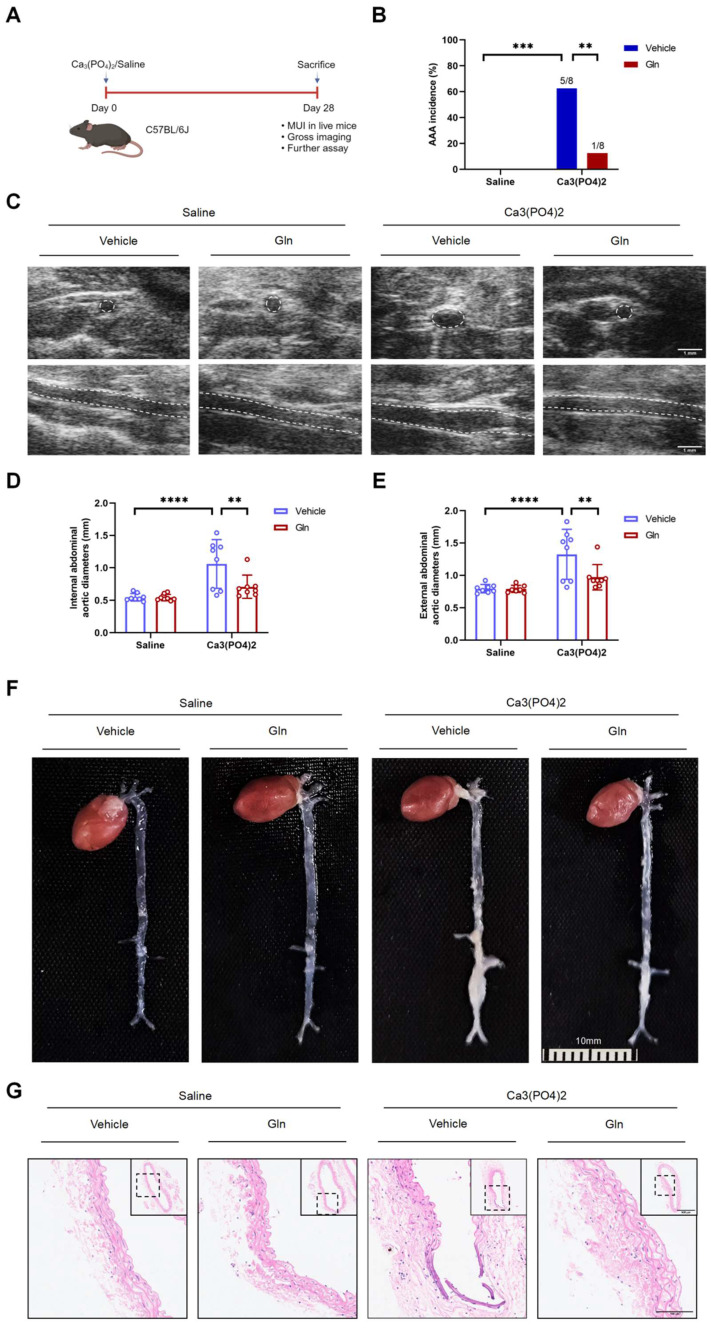
** Glutamine's protective effects above on AngII-induced mouse AAA model were also seen in mouse AAA model by Ca_3_(PO_4_)_2_.** A, Schematic protocol: C57BL/6J mice were isolated and incubated with 0.5 mol/L calcium chloride, followed by phosphate-buffered saline (PBS) to form Ca_3_(PO_4_)_2_ (n=8 per group), 28 days later, C57BL/6J mice were sacrificed. (The image was created with BioRender.com) B, The incidence of Ca_3_(PO_4_)_2_-induced AAA in indicated groups (n=8 per group). Data were analyzed by a Fisher exact test. **P<0.01; ****P<0.0001. C, Representative images of abdominal aortas visualized by MUI using the B mode in indicated groups. D and E, Quantification of the internal diameter of suprarenal abdominal aortas measured by MUI and external diameter by a Digital Vernier Caliper in indicated groups (n=8 per group). Data were analyzed by 2-way ANOVA followed by the Bonferroni post hoc test. **P<0.01; ****P<0.0001. F, Representative images of the macroscopic features of AAA formation in indicated groups. G, Representative images of HE staining in infrarenal abdominal aortas in indicated groups. Data are expressed as mean ± SD. Abbreviations: AngII, angiotensin II; Gln, glutamine; MUI, micro-ultrasound imaging; AAA, abdominal aortic aneurysm; HE staining, hematoxylin and eosin staining.

To further validate the protective role played by glutamine against mouse AAA formation, we constructed mouse AAA model induced by Ca_3_(PO_4_)_2_ (Figure 2A), which has been demonstrated effective in promoting the formation of mouse AAA 3. Consistent with the above experimental results, glutamine treatment also reduced AAA incidence in Ca_3_(PO_4_)_2_-induced mice in comparison with vehicle-treated mice (Figure 2B). Likewise, internal diameter by MUI (Figure 2C) and external diameters by a measuring tape (Figure 2E) of infrarenal abdominal aortas in Ca_3_(PO_4_)_2_-treated mice were decreased by glutamine (Figure 2D and 2F). More complete aortic tissue was seen by glutamine treatment compared with vehicle treatment in Ca_3_(PO_4_)_2_-induced AAA model (Figure 2G).

### Glutamine suppressed elastic fiber degradation and collagen deposition

**Figure 3:**
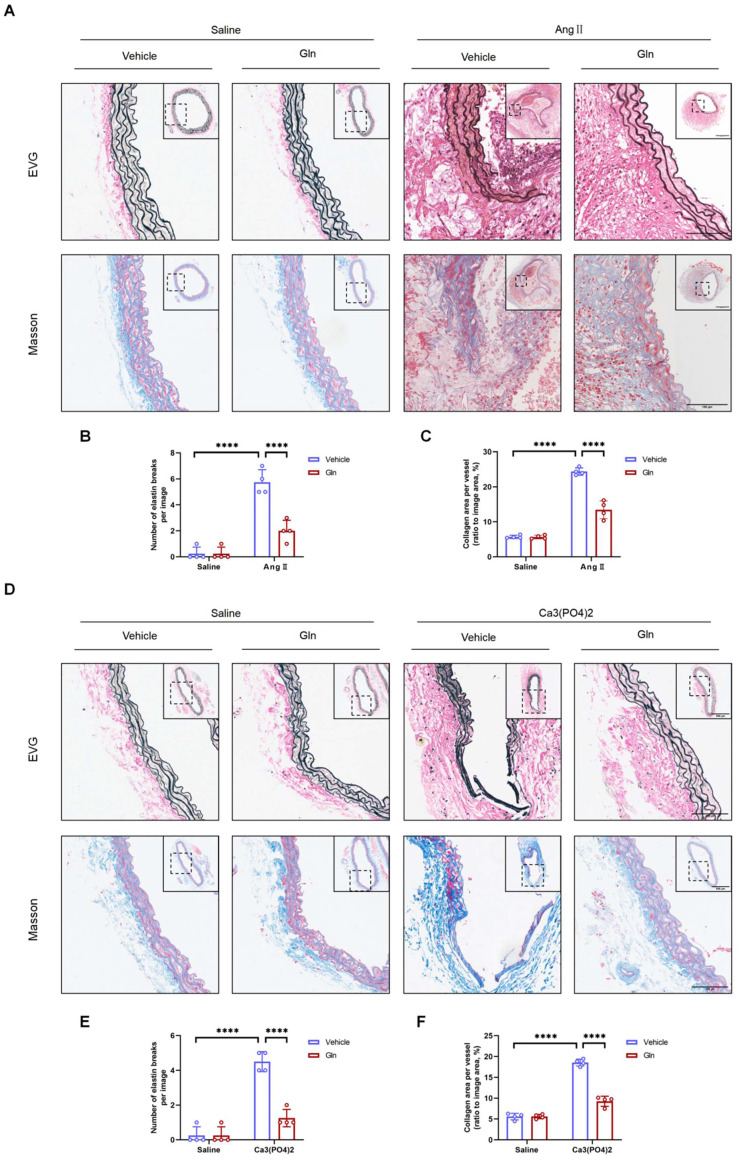
** Glutamine suppressed elastic fiber degradation and collagen deposition.** A, Representative images of morphology staining (EVG staining and MASSON staining) in suprarenal abdominal aortas from ApoE^-/-^ mice in indicated groups. B, Quantification of elastin degradation by counting the number of breaks per image in suprarenal aortic segments based on EVG staining (n=4 per group). ****P<0.0001. C, Quantification of collagen content in suprarenal abdominal aortas, based on Masson Trichrome staining (n=4 per group). ****P<0.0001. D, Representative images of morphology staining (EVG staining and MASSON staining) in infrarenal abdominal aortas from C57BL/6J mice in indicated groups. E, Quantification of elastin degradation by counting the number of breaks per image in infrarenal aortic segments based on EVG staining (n=4 per group). ****P<0.0001. F, Quantification of collagen content in infrarenal abdominal aortas, based on Masson Trichrome staining (n=4 per group). ****P<0.0001. Data were analyzed by two-way ANOVA followed by Bonferroni post-hoc test. Data are expressed as mean ± SD. Abbreviations: AngII, angiotensin II; Gln, glutamine; EVG staining, elastin van Gieson staining.

Previous studies reported that AAA of mice was characterized by elastic fiber degradation and collagen deposition 28. Our current study employed EVG staining and MASSON staining on 3-4 µm thick AAA sections induced by AngII and Ca_3_(PO_4_)_2_ to examine the morphological changes of elastin fiber and histological characteristics. The results showed that elastic fiber degradation was suppressed by glutamine compared with vehicle treatment group in both AngII-induced (Figure 3A and 3B) and Ca_3_(PO_4_)_2_-induced (Figure 3D and 3E) mouse AAA models, likewise, glutamine improved collagen deposition, which is more severe in vehicle-treated, AngII-induced (Figure 3A and 3C) or Ca_3_(PO_4_)_2_-induced (Figure 3D and 3F) mouse aortas.

### Glutamine inhibited MMP activity and expression level

**Figure 4:**
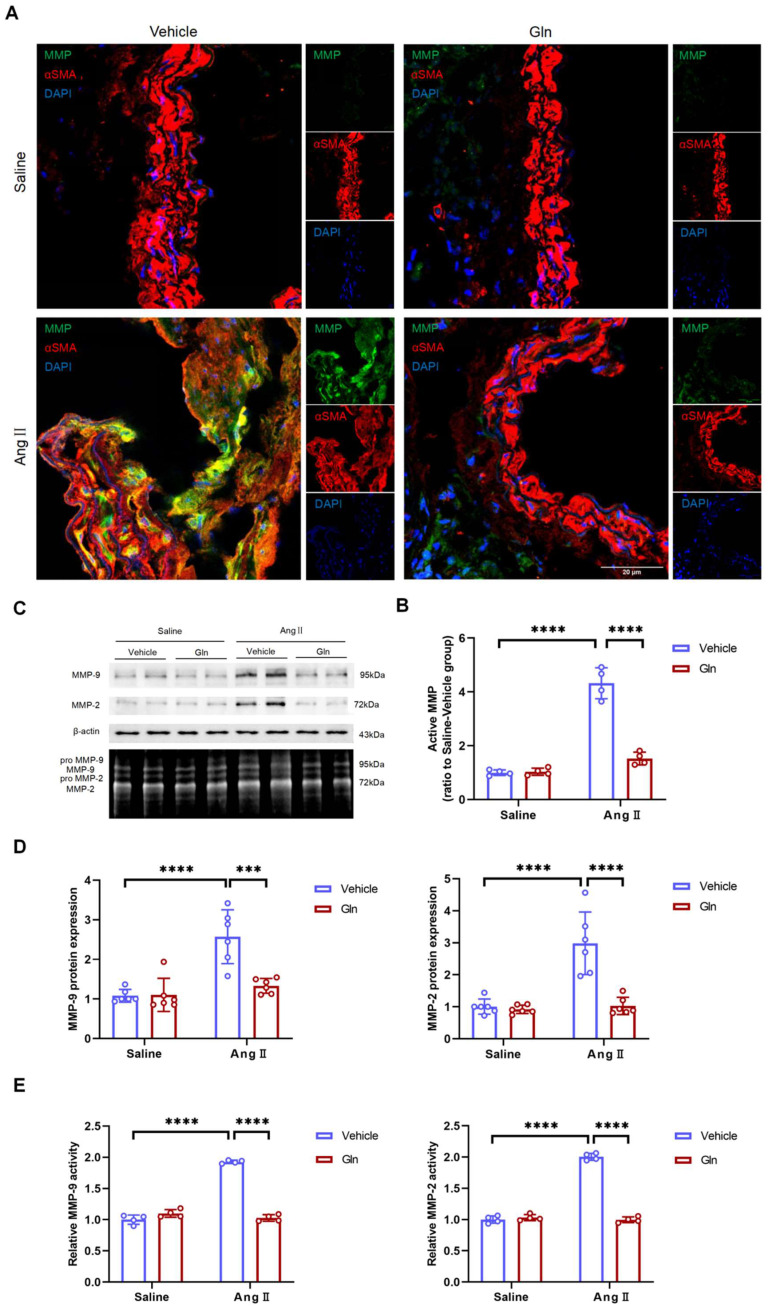
** Glutamine inhibited MMP activity and expression level.** A, Representative images of MMP activity staining in suprarenal abdominal aortas from the indicated groups. B, Quantification of active MMP in indicated groups (n=4 per group). ****P<0.0001. C, MMP-9 protein expression in RAW264.7 and MMP-2 protein expression in MOVAS measured by Western blot and MMP activity in abdominal aortas measured by zymography in indicated groups. D, Left, Quantification of MMP-9 protein expression in RAW264.7 in indicated groups (n=6 per group). Right, Quantification of MMP-2 protein expression in MOVAS in indicated groups (n=6 per group). ***P<0.001; ****P<0.0001. E, Left, Quantification of relative MMP-9 activity in indicated groups (n=4 per group). Right, Quantification of relative MMP-2 activity in indicated groups (n=4 per group). ****P<0.0001. Data were analyzed by two-way ANOVA followed by Bonferroni post-hoc test. Data are expressed as mean ± SD. AngII indicates angiotensin II; Gln, glutamine; MMP, matrix metalloproteinase; MOVAS, mouse aortic smooth muscle cells.

Further, we examined the activity of MMP in suprarenal abdominal aorta, since active MMP promoted degradation of elastic fiber and deposition of collagen 15,16, to validate if the above histomorphological changes were resulted by MMP. Our current work found compared with high MMP activity in vehicle-treated and AngII-induced mouse AAA tissues, glutamine treatment significantly inhibited MMP activity in abdominal aorta of AngII-induced mouse models (Figure 4A and 4B). Gelatin zymography experiment showed that both MMP-2 and MMP-9 activity in AngII-induced abdominal aorta were inhibited by glutamine (Figure 4C and 4E), which was further demonstrated by *in vitro* cells including MOVAS and RAW264.7 experiments, shown with the results of decreased MMP-2 protein secreted by MOVAS and MMP-9 protein by RAW264.7 (Figure 4C and 4D).

### Glutamine attenuated M1 macrophage activation and secretion of IL-6 and TNF-α

**Figure 5:**
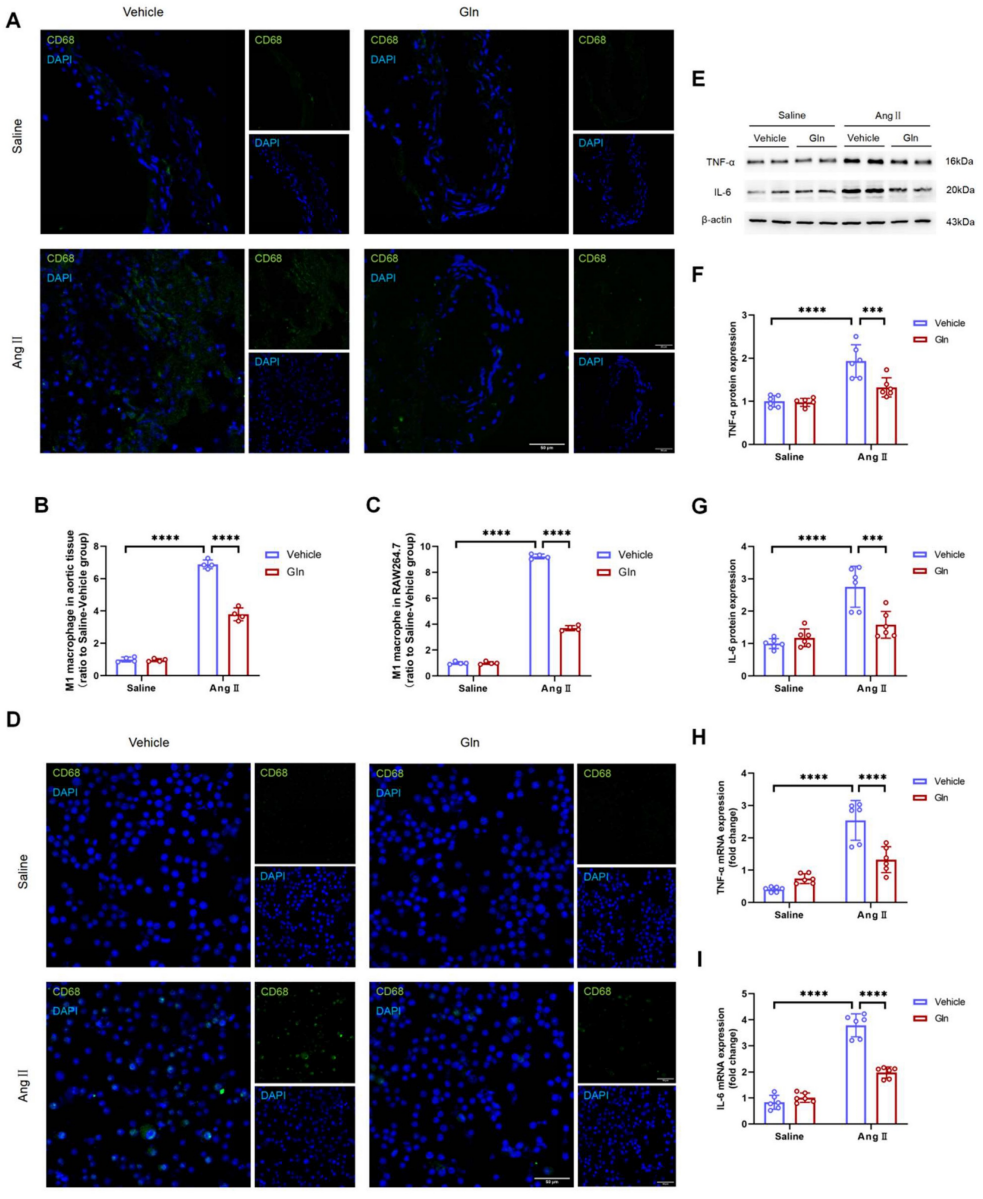
** Glutamine attenuated M1 macrophage activation and secretion of IL-6 and TNF-α.** A and B, Representative images of CD68 staining and quantification of CD68 staining in abdominal aortic tissues (n=4 per group). ****P<0.0001. C and D, Representative images of CD68 staining and quantification of CD68 staining in RAW264.7 (n=4 per group). ****P<0.0001. E, Protein expression of TNF-α and IL-6 in RAW264.7 measured by Western blot in indicated groups. F, Quantification of TNF-α protein expression in indicated groups (n=6 per group). ***P<0.001; ****P<0.0001.G, Quantification of IL-6 protein expression (n=6 per group). ***P<0.001; ****P<0.0001. H, Quantification of TNF-α mRNA expression in RAW264.7 by RT-qPCR in indicated groups (n=6 per group). ****P<0.0001. I, Quantification of IL-6 mRNA expression in RAW264.7 by RT-qPCR in indicated groups (n=6 per group). ****P<0.0001. Data were analyzed by two-way ANOVA followed by the Bonferroni post hoc test. Data are expressed as mean ± SD. AngII indicates angiotensin II; Gln, glutamine; TNF-α, tumor necrosis factor-α; IL-6, interleukin-6; RT-qPCR, real-time quantitative polymerase chain reaction.

To confirm the effect of glutamine on M1 macrophage activation, we conducted the experiment of CD68 protein staining in mouse aortic tissue and RAW264.7, since CD68 is one of the commonly used markers of M1 macrophage. The effect of glutamine on the expression of RAW264.7-derived IL-6 and TNF-α in protein and mRNA level was measured by western blot and RT-qPCR. Our work found AngII-infused aortic tissue (Figure 5A and 5B) and AngII-treated RAW264.7 (Figure 5C and 5D) were characterized with more M1 macrophage activation, compared with saline treatment group, and the activation of M1 macrophage by AngII could be inhibited by glutamine. What's more, the high expression of TNF-α (Figure 5F) and IL-6 (Figure 5G) in protein (Figure 5E) and mRNA (Figure 5H and 5I) in AngII-treated RAW264.7 could also be decreased by glutamine treatment.

### Glutamine repressed the apoptosis of vascular smooth muscle cells and production of ROS

**Figure 6:**
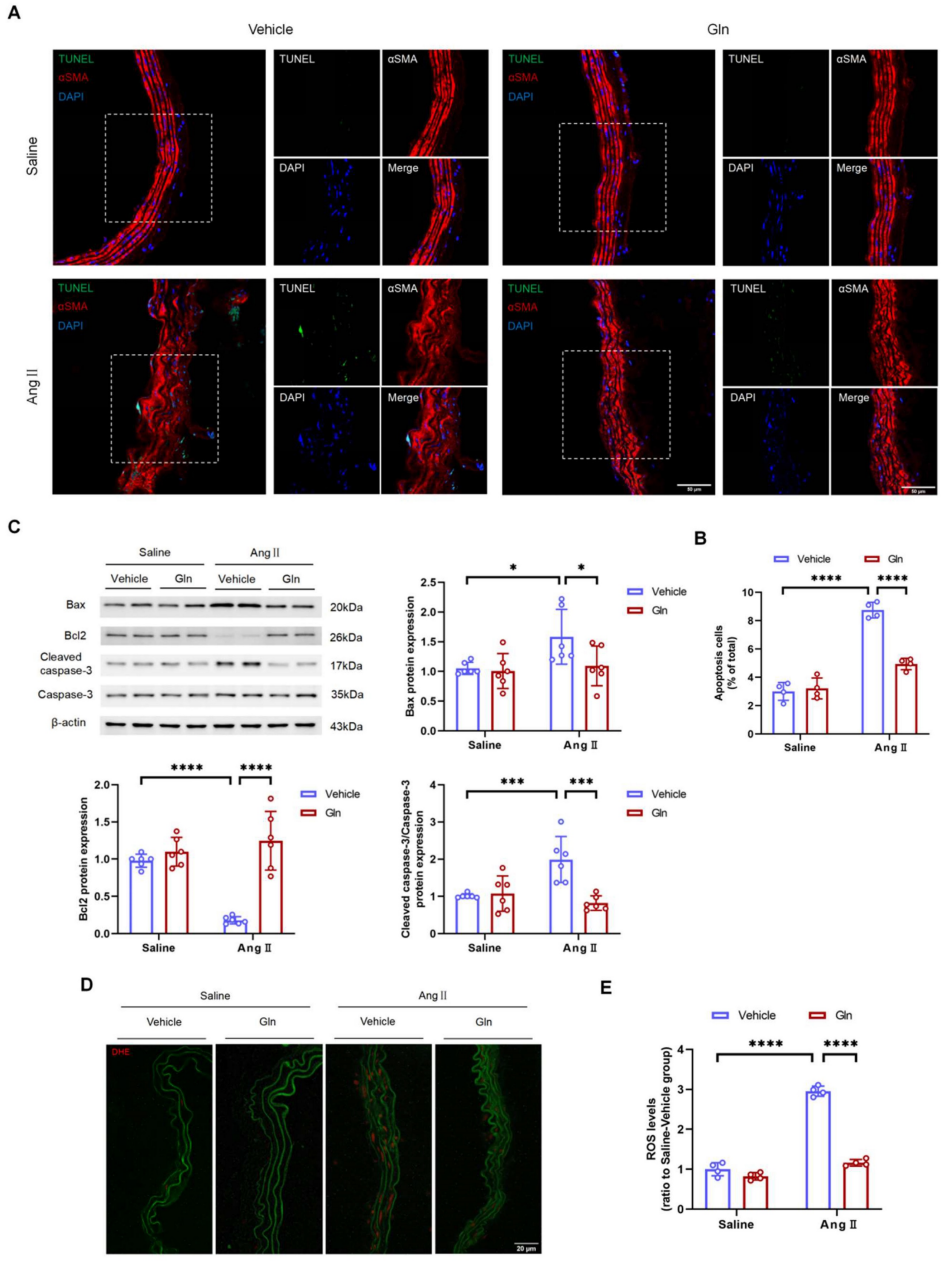
** Glutamine repressed the apoptosis of vascular smooth muscle cells and the production of ROS.** A, Representative images of TUNEL staining in suprarenal abdominal aortas from the indicated groups. B, Quantification of the percentages of TUNEL positive cells in suprarenal abdominal aortas in indicated groups (n=4 per group). ****P<0.0001. C, Quantification of Bax, Bcl2, and cleaved caspase-3/caspase-3 protein expression in MOVAS assayed by Western blot in indicated groups (n=6 per group). *P<0.05; ***P<0.001; ****P<0.0001. D and E, Representative images of in situ DHE staining and quantification of ROS levels in suprarenal abdominal aortas to assess superoxide generation in indicated groups (n=4 per group). ****P<0.0001. Data were analyzed by two-way ANOVA followed by the Bonferroni post hoc test. Data are expressed as mean ± SD. Abbreviations: AngII, angiotensin II; Gln, glutamine; MOVAS, mouse aortic smooth muscle cells; DHE, dihydroethidium; ROS, reactive oxide species.

VSMC apoptosis in physiological conditions is beneficial for normal functions of vessels, however, excessive VSMC apoptosis due to overproduction of MMP and ROS inevitably damages the structure of abdominal aorta, gradually leading to the formation and progression of AAA 17-19. In this study, the TUNEL assay showed that glutamine decreased the level of apoptosis in AngII-infused suprarenal abdominal aortas (Figure 6A and 6B). Likewise, the western blot analysis showed the levels of pro-apoptotic proteins including Bax and Cleaved-caspase-3 expressed by AngII-treated MOVAS were down-regulated, and the level of anti-apoptotic protein Bcl2 expressed by AngII-treated MOVAS was up-regulated, by glutamine treatment (Figure 6C). To confirm the level of oxidative stress, we conducted *in situ* DHE staining on suprarenal abdominal aorta to examine the production of ROS. Our work found AngII-induced mouse AAA groups treated by glutamine were characterized with attenuated production of ROS, compared with vehicle treatment group (Figure 6D and 6E).

### Glutamine protected against mouse abdominal aortic aneurysm through modulating VSMC apoptosis and M1 macrophage activation

**Figure 7:**
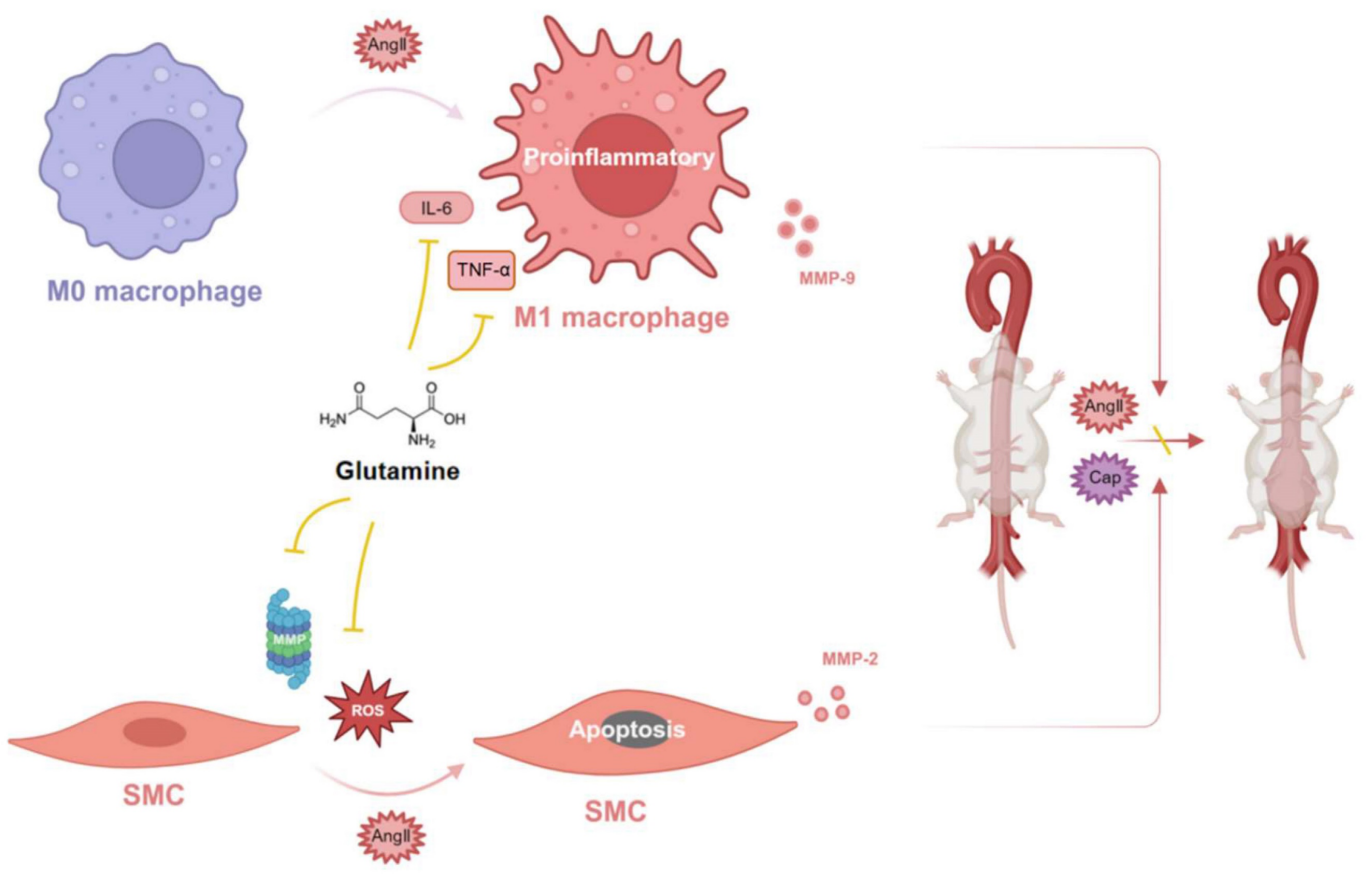
** Glutamine protected against mouse abdominal aortic aneurysm through modulating VSMC apoptosis and M1 macrophage activation.** Glutamine protected against mouse abdominal aortic aneurysm through inhibiting M1 macrophage activation, secretion of IL-6 and TNF-α by M1 macrophage, release of MMP-9 by M1 macrophage and MMP-2 by SMC, as well as apoptosis of vascular SMC by ROS and MMP. Abbreviations: AngII, angiotensin II; CaP, Ca_3_(PO_4_)_2_; ROS, reactive oxide species; MMP, matrix metalloproteinase; TNF-α, tumor necrosis factor-α; IL-6, interleukin-6; SMC, smooth muscle cell. (The image was created with BioRender.com).

Glutamine protected against mouse abdominal aortic aneurysm through inhibiting M1 macrophage activation, secretion of IL-6 and TNF-α by macrophage, release of MMP-9 by RAW264.7 (macrophage) and MMP-2 by MOVAS (SMC), as well as apoptosis of vascular SMC by ROS and MMP (Figure 7).

## Discussion

Currently, AAA remains an important cause of death among adults, causing approximately 200000 deaths per year worldwide, as a result of aortic rupture 29. Previously published randomized-controlled clinical trials failed to demonstrated the limitation of AAA growth by antibiotics or hypotensive drugs 4. To date, clinically demonstrated effective drugs in treating human AAA are still lacking, therefore, this drives a substantial interest in the pathogenesis of AAA. The main way of examining drug effectiveness in AAA or mechanisms involved in AAA pathogenesis is through the use of animal models, particularly mice 3,4. In this study, we employed two animal AAA models, namely AngII-induced and Ca_3_(PO_4_)_2_-induced mouse AAA models, to validate the beneficial role of glutamine in treating mouse AAA. We found decreased generation of ROS and activity of MMP by glutamine in AngII-treated mouse abdominal aortic tissue, meaning that glutamine is effective in attenuating apoptosis of VSMC, inhibiting secretion of inflammatory factors, and maintaining normal structure of aorta, as excessive production of ROS and activity of MMP have been reported to be detrimental factors exacerbating VSMC apoptosis, promoting macrophage polarization, and finally causing the formation and progression of AAA 17,30. Previous studies suggested the involvement of VSMC-derived MMP-2 and macrophage-derived MMP-9 in ECM degradation 31,32; our current study found that glutamine down-regulated the expression of MOVAS-derived MMP-2 and RAW264.7-derived MMP-9 in protein level. Although the therapeutical potential of glutamine for AAA was demonstrated only in the mouse models, this promising data may encourage future clinical trials to assess its effectiveness in attenuating arterial dilatation and remodeling in human patients with AAA.

Previous studies have reported that decreased level of glutamine was associated with dysfunction of immune system 33, elevated ROS level 34, intestinal diseases etc. 35.

Additionally, the level of glutamine was reported to be decreased in AngII-treated group 36, therefore, we conducted this study to validate whether supplementation of glutamine protected mice from AAA, since AngII-induced mouse AAA model is widely used in laboratory. Although some side effects of chronic glutamine supplementation have been discussed 37, it is still widely used as an anti-fatigue amino acid in sport field 38. More importantly, glutamine, as the most abundant free amino acid in human body, is the main substrate utilized by intestinal cells, which means it is necessary for normal function of the digestive system and helpful in managing multiple intestinal diseases 35. Given the exact effects of glutamine supplementation on intestinal diseases, we tried to identified its functions in cardiovascular diseases, aiming to find novel indications. Due to the fact that human AAA lacks drug treatment and supplementation of glutamine is favored by some people, randomized-controlled clinical trials of glutamine are more likely to be accepted by patients with AAA.

Studies from our laboratory found the supplementation of α-KG was effective in improving mitochondrial function and reversing mouse AAA development in both AngII- and Ca_3_(PO_4_)_2-_ induced AAA models 12. Our current study found that glutamine protected against mouse AAA through inhibiting VSMC apoptosis, M1 macrophage activation, and ROS production. We think that the protective effect of glutamine on mouse AAA can be mainly attributed to two reasons, one is the participation of its downstream metabolites like α-KG in the TCA cycle, maintaining the normal function of cells, the other is that glutamine itself serves as a “signal”, promoting its beneficial effects. Cai WF *et al.* reported that glutaminase triggers mitochondrial fusion in a non-enzymatic manner by sensing glutamine availability, deprivation of glutamine itself served as a “signal” that promoted mitochondrial fusion 39. Although glutamine is precursor of α-KG, the protective roles of glutamine in mouse AAA cannot be totally attributed to its conversion into α-KG, we think that its protective effects are better attributed to a combination of itself and its downstream metabolites.

## Conclusion

Our current work revealed the protective effects of glutamine against mouse AAA induced by AngII and Ca_3_(PO_4_)_2_ through modulating VSMC apoptosis and M1 macrophage activation.

## Figures and Tables

**Table 1:** Information of PCR Primers

Gene	Primer	Sequence (5'-3')	Application
*β-actin* (mouse)	Forward	GTGGATCAGCAAGCAGGAGTA	RT-qPCR
	Reverse	GGGTGTAAAACGCAGCTCAGTA	RT-qPCR
*IL-6* (mouse)	Forward	CTGCAAGAGACTTCCATCCAG	RT-qPCR
	Reverse	AGTGGTATAGACAGGTCTGTTGG	RT-qPCR
*TNF-α* (mouse)	Forward	CAGGCGGTGCCTATGTCTC	RT-qPCR
	Reverse	CGATCACCCCGAAGTTCAGTAG	RT-qPCR

## Abbreviations

Gln: Glutamine
AAA: Abdominal aortic aneurysm
AngII: Angiotensin II
MUI: Micro-ultrasound imaging
MMP: Matrix metalloproteinase
ROS: Reactive oxide species
TNF-α: Tumor necrosis factor-α
IL-6: Interleukin-6
SMC: Smooth muscle cell
EVG staining: Elastin van Gieson staining
HE staining: Hematoxylin and eosin staining
DHE: Dihydroethidium
MOVAS: Mouse aortic smooth muscle cells

## References

[1] Chaikof EL, Dalman RL, Eskandari MK, Jackson BM, Lee WA, Mansour MA (2018). The Society for Vascular Surgery practice guidelines on the care of patients with an abdominal aortic aneurysm. J Vasc Surg.

[2] Haque K, Bhargava P (2022). Abdominal Aortic Aneurysm. Am Fam Physician.

[3] Golledge J (2019). Abdominal aortic aneurysm: update on pathogenesis and medical treatments. Nat Rev Cardiol.

[4] Golledge J, Moxon JV, Singh TP, Bown MJ, Mani K, Wanhainen A (2020). Lack of an effective drug therapy for abdominal aortic aneurysm. J Intern Med.

[5] Thomas K, Zondler L, Ludwig N, Kardell M, Lüneburg C, Henke K (2022). Glutamine prevents acute kidney injury by modulating oxidative stress and apoptosis in tubular epithelial cells. JCI Insight.

[6] Petry ÉR, Dresch DdF, Carvalho C, Medeiros PC, Rosa TG, de Oliveira CM (2019). Oral glutamine supplementation attenuates inflammation and oxidative stress-mediated skeletal muscle protein content degradation in immobilized rats: Role of 70 kDa heat shock protein. Free Radic Biol Med.

[7] Shrestha N, Chand L, Han MK, Lee SO, Kim CY, Jeong YJ (2016). Glutamine inhibits CCl4 induced liver fibrosis in mice and TGF-β1 mediated epithelial-mesenchymal transition in mouse hepatocytes. Food Chem Toxicol.

[8] Durante W (2019). The Emerging Role of l-Glutamine in Cardiovascular Health and Disease. Nutrients.

[9] Salabei JK, Lorkiewicz PK, Holden CR, Li Q, Hong KU, Bolli R (2015). Glutamine Regulates Cardiac Progenitor Cell Metabolism and Proliferation. Stem Cells.

[10] Zhang C-Y, Hu Y-C, Zhang Y, Ma W-D, Song Y-F, Quan X-H (2021). Glutamine switches vascular smooth muscle cells to synthetic phenotype through inhibiting miR-143 expression and upregulating THY1 expression. Life Sci.

[11] Ma W, Heianza Y, Huang T, Wang T, Sun D, Zheng Y (2018). Dietary glutamine, glutamate and mortality: two large prospective studies in US men and women. Int J Epidemiol.

[12] Sun L-Y, Lyu Y-Y, Zhang H-Y, Shen Z, Lin G-Q, Geng N (2022). Nuclear Receptor NR1D1 Regulates Abdominal Aortic Aneurysm Development by Targeting the Mitochondrial Tricarboxylic Acid Cycle Enzyme Aconitase-2. Circulation.

[13] Lagranha CJ, Hirabara SM, Curi R, Pithon-Curi TC (2007). Glutamine supplementation prevents exercise-induced neutrophil apoptosis and reduces p38 MAPK and JNK phosphorylation and p53 and caspase 3 expression. Cell Biochem Funct.

[14] Rombouts KB, van Merrienboer TAR, Ket JCF, Bogunovic N, van der Velden J, Yeung KK (2022). The role of vascular smooth muscle cells in the development of aortic aneurysms and dissections. Eur J Clin Invest.

[15] Chang Z, Zhao G, Zhao Y, Lu H, Xiong W, Liang W (2020). BAF60a Deficiency in Vascular Smooth Muscle Cells Prevents Abdominal Aortic Aneurysm by Reducing Inflammation and Extracellular Matrix Degradation. Arterioscler Thromb Vasc Biol.

[16] Stepien KL, Bajdak-Rusinek K, Fus-Kujawa A, Kuczmik W, Gawron K (2022). Role of Extracellular Matrix and Inflammation in Abdominal Aortic Aneurysm. Int J Mol Sci.

[17] Atkinson G, Bianco R, Di Gregoli K, Johnson JL (2023). The contribution of matrix metalloproteinases and their inhibitors to the development, progression, and rupture of abdominal aortic aneurysms. Front Cardiovasc Med.

[18] Chen Q, Wang Q, Zhu J, Xiao Q, Zhang L (2018). Reactive oxygen species: key regulators in vascular health and diseases. Br J Pharmacol.

[19] Sánchez-Infantes D, Nus M, Navas-Madroñal M, Fité J, Pérez B, Barros-Membrilla AJ (2021). Oxidative Stress and Inflammatory Markers in Abdominal Aortic Aneurysm. Antioxidants (Basel).

[20] Yuan Z, Lu Y, Wei J, Wu J, Yang J, Cai Z (2020). Abdominal Aortic Aneurysm: Roles of Inflammatory Cells. Front Immunol.

[21] Raffort J, Lareyre F, Clément M, Hassen-Khodja R, Chinetti G, Mallat Z (2017). Monocytes and macrophages in abdominal aortic aneurysm. Nat Rev Cardiol.

[22] Jabłońska A, Zagrapan B, Neumayer C, Eilenberg W, Scheuba A, Brostjan C (2021). Polymorphisms in the IL-6 and TNF-α gene are associated with an increased risk of abdominal aortic aneurysm. Int J Cardiol.

[23] Batra R, Suh MK, Carson JS, Dale MA, Meisinger TM, Fitzgerald M (2018). IL-1β (Interleukin-1β) and TNF-α (Tumor Necrosis Factor-α) Impact Abdominal Aortic Aneurysm Formation by Differential Effects on Macrophage Polarization. Arterioscler Thromb Vasc Biol.

[24] O'Leary R, Penrose H, Miyata K, Satou R (2016). Macrophage-derived IL-6 contributes to ANG II-mediated angiotensinogen stimulation in renal proximal tubular cells. Am J Physiol Renal Physiol.

[25] Gao R, Guo W, Fan T, Pang J, Hou Y, Feng X (2022). Phosphodiesterase 4D contributes to angiotensin II-induced abdominal aortic aneurysm through smooth muscle cell apoptosis. Exp Mol Med.

[26] Emeto TI, Moxon JV, Au M, Golledge J (2016). Oxidative stress and abdominal aortic aneurysm: potential treatment targets. Clin Sci (Lond).

[27] Maguire EM, Pearce SWA, Xiao R, Oo AY, Xiao Q (2019). Matrix Metalloproteinase in Abdominal Aortic Aneurysm and Aortic Dissection. Pharmaceuticals (Basel).

[28] Jana S, Hu M, Shen M, Kassiri Z (2019). Extracellular matrix, regional heterogeneity of the aorta, and aortic aneurysm. Exp Mol Med.

[29] Sampson UKA, Norman PE, Fowkes FGR, Aboyans V, Yanna S, Harrell FE (2014). Global and regional burden of aortic dissection and aneurysms: mortality trends in 21 world regions, 1990 to 2010. Glob Heart.

[30] Liu C, Hu F, Jiao G, Guo Y, Zhou P, Zhang Y (2022). Dental pulp stem cell-derived exosomes suppress M1 macrophage polarization through the ROS-MAPK-NFκB P65 signaling pathway after spinal cord injury. J Nanobiotechnology.

[31] Longo GM, Xiong W, Greiner TC, Zhao Y, Fiotti N, Baxter BT (2002). Matrix metalloproteinases 2 and 9 work in concert to produce aortic aneurysms. J Clin Invest.

[32] Galis ZS, Khatri JJ (2002). Matrix metalloproteinases in vascular remodeling and atherogenesis: the good, the bad, and the ugly. Circ Res.

[33] Durante W (2023). Glutamine Deficiency Promotes Immune and Endothelial Cell Dysfunction in COVID-19. Int J Mol Sci.

[34] Gwangwa MV, Joubert AM, Visagie MH (2019). Effects of glutamine deprivation on oxidative stress and cell survival in breast cell lines. Biol Res.

[35] Kim M-H, Kim H (2017). The Roles of Glutamine in the Intestine and Its Implication in Intestinal Diseases. Int J Mol Sci.

[36] Chao de la Barca JM, Richard A, Robert P, Eid M, Fouquet O, Tessier L (2022). Metabolomic Profiling of Angiotensin-II-Induced Abdominal Aortic Aneurysm in Ldlr-/- Mice Points to Alteration of Nitric Oxide, Lipid, and Energy Metabolisms. Int J Mol Sci.

[37] Holecek M (2013). Side effects of long-term glutamine supplementation. JPEN J Parenter Enteral Nutr.

[38] Coqueiro AY, Rogero MM, Tirapegui J (2019). Glutamine as an Anti-Fatigue Amino Acid in Sports Nutrition. Nutrients.

[39] Cai W-F, Zhang C, Wu Y-Q, Zhuang G, Ye Z, Zhang C-S (2018). Glutaminase GLS1 senses glutamine availability in a non-enzymatic manner triggering mitochondrial fusion. Cell Res.

